# A Pilot Surveillance Program to Identify Emerging SARS-CoV-2 Variants in International Travelers Arriving at a Major International Airport: Prospective Cohort Evaluation

**DOI:** 10.2196/96414

**Published:** 2026-11-06

**Authors:** Eric M Foote, Ellen L Bouchard, Elizabeth F Baylis, Shiffen Getabecha, Phacharee Arunleung, Maria Salas, Gautham Gautham, Christina Morales, Debra A Wadford, Kathleen Jacobson

**Affiliations:** 1 Stanford University, Center for Academic Medicine, 453 Quarry Road, Palo Alto, CA, 94304, United States, 1 (650) 723-5711; 2 California Department of Public Health, Richmond, CA, United States

**Keywords:** traveler SARS-CoV-2 genomic surveillance, pandemic preparedness, whole-genome sequencing, COVID-19 antigen tests, international airport screening

## Abstract

**Background:**

Testing arriving international travelers for COVID-19 paired with whole-genome sequencing (WGS) may identify new introductions of SARS-CoV-2 variants of concern in California, enabling a timely public health response. Traveler genomic surveillance may be a way to improve gaps in worldwide disease genomic surveillance.

**Objective:**

This paper aimed to describe a pilot surveillance program to identify SARS-CoV-2 variants in arriving international travelers at San Francisco International Airport (SFO).

**Methods:**

The California Department of Public Health implemented a pilot program at SFO to voluntarily test arriving international travelers for COVID-19 from June 2021 to November 2021 to identify SARS-CoV-2 variants. Arriving international travelers were offered self-collect COVID-19 antigen tests upon arrival at SFO. Travelers with positive COVID-19 antigen tests or with COVID-19 symptoms were recommended to self-collect and mail an additional specimen for diagnostic molecular testing. WGS of SARS-CoV-2 was performed on RNA extracts from positive tests to identify variants.

**Results:**

In total, 2.1% (19,008/892,558) of all arriving international travelers at SFO from 133 different countries of departure enrolled in the program. The COVID-19 test positivity among this group was 0.7% (33/5038), and the weekly test positivity range, 0% to 1.5%, was lower than the weekly test positivity across the state of California during the same time frame. Arriving international travelers departing from Turkey (3/145, 2.1%), Mexico (7/602, 1.2%), and the United Kingdom (6/626, 1.0%) had the highest test positivity. Of the 14 positive molecular tests, 10 (71.4%) were sequenced, and all were found to belong to the SARS-CoV-2 Delta B.1.617.2 lineage, the most prevalent SARS-CoV-2 variant in California and worldwide at the time.

**Conclusions:**

Voluntary testing of arriving international travelers paired with WGS was operationally and logistically feasible. Although the number of sequenced positive COVID-19 specimens in this program was limited, the lessons learned from this pilot informed improvements to subsequent iterations of traveler viral genomic surveillance in California and highlighted the potential value of traveler viral genomic surveillance to support early identification and characterization of emerging infectious diseases entering a region.

## Introduction

Since the beginning of the COVID-19 pandemic, arriving international travelers have posed a risk of introducing new SARS-CoV-2 variants into the United States [1,2]. Many variants of concern that became dominant variants within the United States originated outside of the country [1]. Testing and genomic characterization of SARS-CoV-2 from travelers as they arrive in the United States may identify new variants of concern, enabling a timely and improved public health response.

In June 2021, worldwide rates of vaccination against COVID-19 were low; approximately 5% of the global population had completed an initial COVID-19 vaccine series [3]. At the same time, international travel to the United States was on the rise. The volume of airline passengers arriving in the United States via San Francisco International Airport (SFO) increased from a low of 2000 passengers per week in April 2020 to 35,000 passengers per week in June 2021 and 46,000 passengers per week in November 2021 [4].

In January 2021, the Centers for Disease Control and Prevention (CDC) mandated that arriving international travelers to the United States test negative for COVID-19 within 3 days of their flight’s departure [5]. It was estimated that predeparture testing could miss detection of up to 64% of arriving international travelers who would go on to become infectious with COVID-19 after arrival [6]. The CDC also recommended that arriving international travelers self-test for COVID-19 within 3 to 5 days after arrival, which, when combined with predeparture testing, could identify 70% of infectious days across all arriving international travelers [5,6]. However, lack of COVID-19 test availability, the high cost of tests, and the public’s unfamiliarity with self-testing all served as potential barriers for arriving international travelers to follow these guidelines. Furthermore, at the time, there was no system to identify SARS-CoV-2 variant status via whole-genome sequencing (WGS) among arriving international travelers, nor was there a system to understand vaccine uptake among travelers arriving in California. Beginning on October 25, 2021, noncitizens who were nonimmigrants were required to be fully vaccinated against COVID-19 to travel to the United States [7].

In 2021, there were large gaps in SARS-CoV-2 WGS worldwide, and it was minimal in many countries [8]. More than 50% of low- to middle-income countries had less than 0.5% of SARS-CoV-2 cases sequenced, leading to large gaps in genomic surveillance for much of the world [8]. It was not clear whether the lack of diversity in SARS-CoV-2 variants identified at this stage was due to a lack of diversity of variants or a lack of sequencing. There was a need to improve gaps in SARS-CoV-2 genomic surveillance, particularly in California, the site of 2 major international airports where travelers could bring novel variants of concern to California from places with minimal WGS.

We hypothesized that targeted testing and WGS of positive specimens from arriving international travelers would be an efficient strategy to identify emerging SARS-CoV-2 variants and augment the existing viral genomic surveillance system in California. The program could help close gaps in the global SARS-CoV-2 genomic surveillance by identifying SARS-CoV-2 variants in travelers arriving from countries with lower sequencing coverage. Between June 2021 and November 2021, the California Department of Public Health (CDPH) implemented a pilot viral genomic surveillance program at SFO to test arriving international travelers for COVID-19 and identify SARS-CoV-2 variants via WGS. The pilot was also an opportunity to establish a partnership with SFO for ongoing COVID-19 testing and SARS-CoV-2 genomic surveillance. In this paper, we present an evaluation of this pilot program and describe its potential operational and logistical feasibility as a part of California’s SARS-CoV-2 surveillance strategy.

## Methods

### Study Design

This was a prospective study evaluating a 2-phased pilot program consisting of COVID-19 diagnostic testing and SARS-CoV-2 genomic sequencing of arriving international travelers implemented at SFO from June 2021 to November 2021.

### Setting

The pilot program took place at the international terminal of SFO, which services routes between 50 international cities and 32 countries. During the pilot period from June 29, 2021, to November 16, 2021, approximately 892,558 passengers deplaned from international flights at SFO [4].

### Ethical Considerations

This surveillance protocol was reviewed by the Committee for the Protection of Human Subjects, California Health and Human Services Agency (project number 2021-180), and determined to not constitute human participant research. Written informed consent for diagnostic COVID-19 testing was obtained from participants at enrollment. Arriving international traveler participation was entirely voluntary, and staff at the airport verbally explained the purpose of this pilot program, its procedures, and the option to decline participation at any point. Participants were informed that their test results and associated data would be used for public health purposes. Participants were not compensated. All data were collected and managed using HIPAA (Health Insurance Portability and Accountability Act)–compliant software and were accessible only to authorized personnel involved in the public health response.

### Participants

#### Pilot Phase 1 Eligibility

Travelers were eligible to participate in the first phase of the pilot program if they were aged at least 2 years and their country of departure was outside of the United States.

#### Pilot Phase 2 Eligibility

Travelers were eligible to participate in the second phase of the pilot program if they were aged at least 18 years and their country of departure was outside of the United States.

#### Recruitment

Participation was voluntary. Announcements were made over the airport public announcement system to inform arriving international travelers of the option to receive free COVID-19 test kits as they exited the international terminal. Signs were placed in the international terminal to inform arriving international travelers about the program as they approached the test site. Trained program staff asked arriving international travelers if they were interested in participating in the pilot program as they passed by the program station, located at the end of the walkway as travelers exit the international arrival area (Figure 1). Recruitment materials were in English.

**Figure 1.**
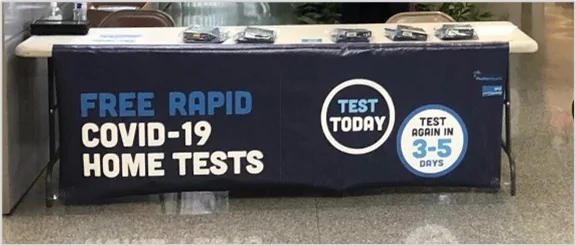
California Department of Public Health COVID-19 testing site at San Francisco International Airport. Arriving international travelers were asked by program staff if they were interested in participating in a voluntary COVID-19 testing program and enrolled as they exited the international terminal from June 29, 2021, to November 16, 2021. The test site operated approximately 8 hours per day and 5 days per week.

#### Enrollment

Participating arriving international travelers provided informed consent for testing, country of departure, vaccination status, and arrival city within California. Enrollment materials were in 10 languages. “Fully vaccinated” was defined for travelers for the purposes of this pilot as having completed an initial series of an approved World Health Organization vaccine [9]. If a passenger reported that they had not completed the initial vaccination series, they were offered the opportunity to receive a vaccine at the SFO medical clinic.

### Pilot Phase 1 Study Design

Phase 1 took place from June 29, 2021, to October 8, 2021 (approximately 3 months; Table 1). Test site staff offered arriving international travelers 2 free BinaxNOW COVID-19 Antigen Self-Tests (Abbott) and a Color COVID-19 Self-Swab Collection Kit (Color Health, Inc) as they passed by the program table (Figures 1 and 2) [10,11]. The test site operated approximately 8 hours per day and 5 days per week. Participating arriving international travelers were instructed via flyer, email, and SMS text message to get tested at home on the day of arrival using a BinaxNOW COVID-19 Antigen Self-Test following the instructions in the box and report their results online via a web-based reporting software (PrimaryHealth) [11]. Participating arriving international travelers received email and SMS text message reminders to take the second antigen test at home 3 to 5 days after arrival per CDC guidelines [2]. If a participating arriving international traveler tested positive, or if they tested negative but had symptoms of COVID-19, they were recommended to self-collect an anterior nasal sample for molecular testing. Participants packaged the self-collected dry swab specimen and mailed it to the Color Health laboratory. Samples had to be received by the laboratory within 56 hours after collection and were tested at Color Health laboratory via the Color SARS-CoV-2 RT-LAMP Diagnostic Assay (Color Health, Inc) [11,12]. Positive molecular tests were then sequenced by the CDPH Viral and Rickettsial Disease Laboratory to determine the SARS-CoV-2 lineage as part of California COVIDNet [13]. All passengers who tested positive for COVID-19 with an antigen test or a molecular test were recommended to isolate per CDPH guidance [12].

**Table 1.** Description of the arriving international traveler COVID-19 testing and SARS-CoV-2 variant identification pilot program at San Francisco International Airport (June 29, 2021-November 16, 2021).

	Phase 1	Phase 2
COVID-19 test	2 home antigen tests and a home self-collection confirmatory molecular test	Home self-collection molecular test mailed to laboratory
Tests, n	3	1
Testing cadence	Day of arrival and 3-5 d after arrival	3-5 d after arrival or immediately if symptoms presented
Test turnaround time	15-30 min	1-3 d
Symptoms recorded at time of initial test	No	Yes
Confirmatory test	Home self-collection molecular test	None
Confirmatory test indications	Antigen test positive, or antigen test negative and symptoms present	—^a^
All positive molecular tests	Whole-genome sequencing to identify SARS-CoV-2 variant	Whole-genome sequencing to identify SARS-CoV-2 variant

a Not applicable.

**Figure 2.**
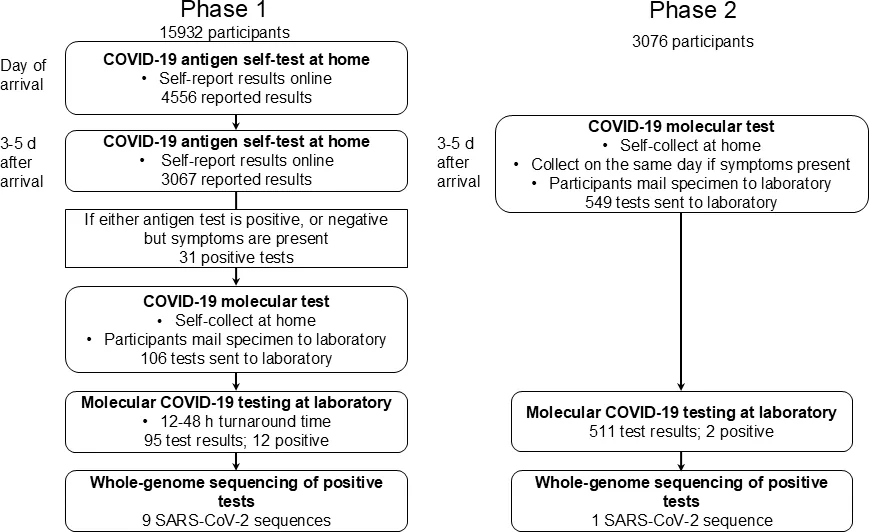
Flow diagram for the arriving international traveler COVID-19 testing and SARS-CoV-2 variant identification pilot surveillance program.

### Pilot Phase 2 Study Design

Phase 2 took place from October 12, 2021, to November 16, 2021 (Table 1 and Figure 2). As with phase 1, the SFO test site for phase 2 operated for 8 hours a day, 5 days per week, and staff offered arriving international travelers a Color COVID-19 Self-Swab Collection Kit as they passed by the staff table (Figure 1). Participating arriving international travelers were instructed via a flyer to get tested at home 3 to 5 days after arrival using the Color COVID-19 Self-Swab Collection Kit following the instructions provided [10,14]. If the participants had symptoms of COVID-19, they were recommended to self-collect a sample for molecular testing immediately. Self-collected anterior nasal dry swabs were tested at the Color Health laboratory via the Color SARS-CoV-2 RT-LAMP Diagnostic Assay [14], as described above [10,14]. A major difference of phase 2 was that participants self-reported whether they had symptoms of COVID-19 at the time of testing, whereas in phase 1, they did not.

### Sample Processing and WGS

Nucleic acid extraction of molecular specimen swabs was performed at the Color Health laboratory. Nucleic acid extracts frozen at –70 °C were shipped to the CDPH Viral and Rickettsial Disease Laboratory on dry ice for WGS. Specimens were tested using an in-house real-time reverse-transcription polymerase chain reaction test to determine cycle threshold (Ct) values. Specimens with Ct <33 were sequenced using either the Illumina MiSeq system according to a published protocol or the Clear Dx SARS-CoV-2 test (Clear Labs) on the GridION sequencer (Oxford Nanopore Technologies) [15-17].

### Data Analysis

We provide descriptive analysis of the data collected in the pilot programs. Statewide COVID-19 test positivity and vaccination data were sourced from the CDPH Health and Human Services Agency Open Data Portal [18]. International vaccination data were sourced from Our World in Data [3]. Arriving international traveler data were sourced from public SFO traveler data [4].

## Results

### Participant Demographics

In total, 19,008 arriving international travelers enrolled in the pilot (phase 1: n=15,932, 83.8%; phase 2: n=3076, 16.2%; Table 2 and Figure 2). Arriving international travelers of all ages (median 42.3, IQR 31.5-55.3 years) arrived from 133 different countries of departure (Table 2). We estimate that 2.1% (19,008/892,558) of all international travelers arriving at SFO participated during the pilot period (Table 2). The most frequently listed countries of departure were Mexico, France, Germany, the United Kingdom, and South Korea, which cumulatively accounted for 39.8% (6924/17,418) of the arriving international travelers (Table 2).

**Table 2.** Demographics and pilot results for the arriving international traveler COVID-19 testing and SARS-CoV-2 variant identification pilot program at San Francisco International Airport (SFO; June 29, 2021-November 16, 2021).^a^

Overall participant demographics	Values
Participants	19,008
SFO arriving international travelers participating in the pilot, n/N (%)	19,008/892,558 (2.1)
Phase 1	15,932/646,145 (2.5)
Phase 2	3076/246,413 (1.2)
Age (y), median (IQR)	42.3 (31.5-55.3)
Countries, n	133
Top 3 countries of departure, n/N (%)	
Mexico	2079/17,418 (11.9)
France	1453/17,418 (8.3)
Germany	1398/17,418 (8.0)
Participants who completed an initial vaccination series, n/N (%)	14,335/17,418 (82.3)
Participants with test results, n/N (%)	5038/19,008 (26.5)
Participant COVID-19 test positivity, n/N (%)	33/5038 (0.7)
Phase 1 antigen test results	
Participants, n	15,932
Submitted COVID-19 antigen tests with results, n/N (%)	7474/7499 (99.7)
Participants who submitted at least 1 antigen test, n/N (%)	4556/15,932 (28.6)
Participants who submitted at least 2 antigen tests, n/N (%)	3067/15,932 (19.3)
COVID-19 antigen test positivity, n/N (%)	31/7474 (0.4)
Participant test positivity, n/N (%)	31/4527 (0.7)
Phase 1 molecular test results	
Participants who submitted COVID-19 molecular tests, n	106
Submitted COVID-19 molecular tests with a positive or negative result, n/N (%)	95/106 (89.6)
COVID-19 molecular test positivity, n/N (%)	12/95 (12.6)
COVID-19 molecular test positivity among those with COVID-19 symptoms, n/N (%)	12/66 (18.2)
Participants who had COVID-19 symptoms among those who tested positive, n/N (%)	12/12 (100)
Participants who tested positive for COVID-19 with an antigen test who submitted a COVID-19 molecular test, n/N (%)	10/30 (33.3)
SARS-CoV-2 sequences generated amongst positive molecular tests, n/N (%)	9/12 (75.0)
Phase 2 results	
Participants, n/N (%)	3076
Participants who submitted a COVID-19 molecular test, n/N (%)	549/3076 (17.8)
Submitted COVID-19 molecular tests with results, n/N (%)	511/549 (93.1)
COVID-19 molecular test positivity, n/N (%)	2/511 (0.4)
Participants with test results with COVID-19 symptoms, n/N (%)	45/511 (8.8)
Participants with COVID-19 symptoms among those who tested positive, n/N (%)	2/2 (100)
COVID-19 molecular test positivity among those with COVID-19 symptoms, n/N (%)	2/45 (4.4)
Participants with COVID-19 symptoms among those who tested negative, n/N (%)	43/509 (8.4)
SARS-CoV-2 sequences generated, amongst positive molecular tests, n/N (%)	1/2 (50)

a Total number of participating arriving international travelers was 19,008.

During the program, 91.6% (17,418/19,008) of the arriving international travelers reported their COVID-19 vaccination status, and 82.3% (14,335/17,418) reported completing an initial COVID-19 vaccination series. Rates of reported complete initial vaccination series increased over the course of the pilot and were generally higher than those for state of California residents (Figure 3 [18]).

**Figure 3.**
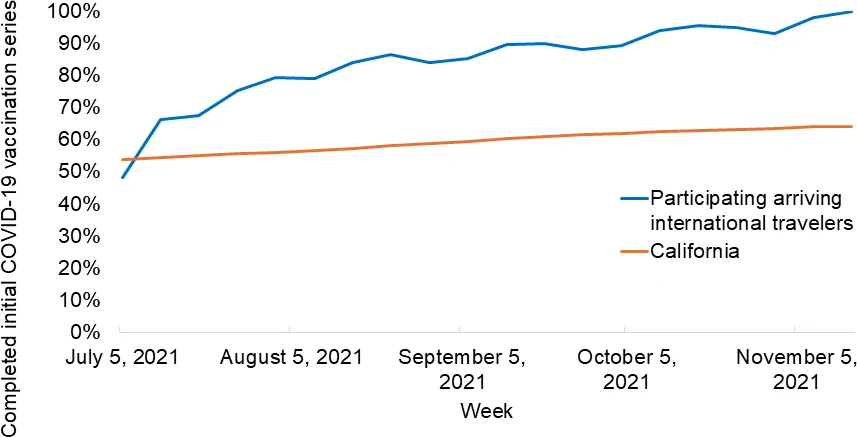
COVID-19 vaccination status for participating arriving international travelers and the general California population. The graph depicts the percentage of the California population (orange line) and of participating arriving international travelers (blue line) at San Francisco International Airport who reported completing the full initial COVID-19 vaccination series by week from June 29, 2021, to November 16, 2021 [18].

Arriving international traveler–reported initial vaccination series completion rates varied by country of departure. Participants who departed from Japan and Taiwan reported the lowest completion rates (Multimedia Appendix 1). Participants who departed from Switzerland and Croatia reported the highest completion rates (Multimedia Appendix 1). The percentage of participating arriving international travelers who reported completing their initial vaccination series increased over time (Multimedia Appendix 2). The reported initial vaccination series completion rate for participating arriving international travelers was generally higher than the rate for their country of departure (Multimedia Appendix 3) for the countries we examined.

### COVID-19 Test Results

During the pilot, 0.7% (33/5038) of participating arriving international travelers with test results tested positive (Table 2). In phase 1, a total of 0.7% (31/4527) of participating arriving international travelers tested positive. In phase 2, a total of 0.4% (2/511) participating arriving international travelers tested positive. The percentage of positivity of COVID-19 tests among airport program participants ranged from 0% to 1.5% weekly. Test positivity of participating arriving international travelers was lower than the test positivity rate across the state of California during the same time frame, which ranged from 1.8% to 7.2% weekly (Figure 4 [18]). Test positivity varied by country of departure (Figure 5). Participating arriving international travelers departing from Turkey (3/145, 2.1%), Mexico (7/602, 1.2%), and the United Kingdom (6/626, 1.0%) had the highest COVID-19 test positivity during the pilot (Figure 5).

**Figure 4.**
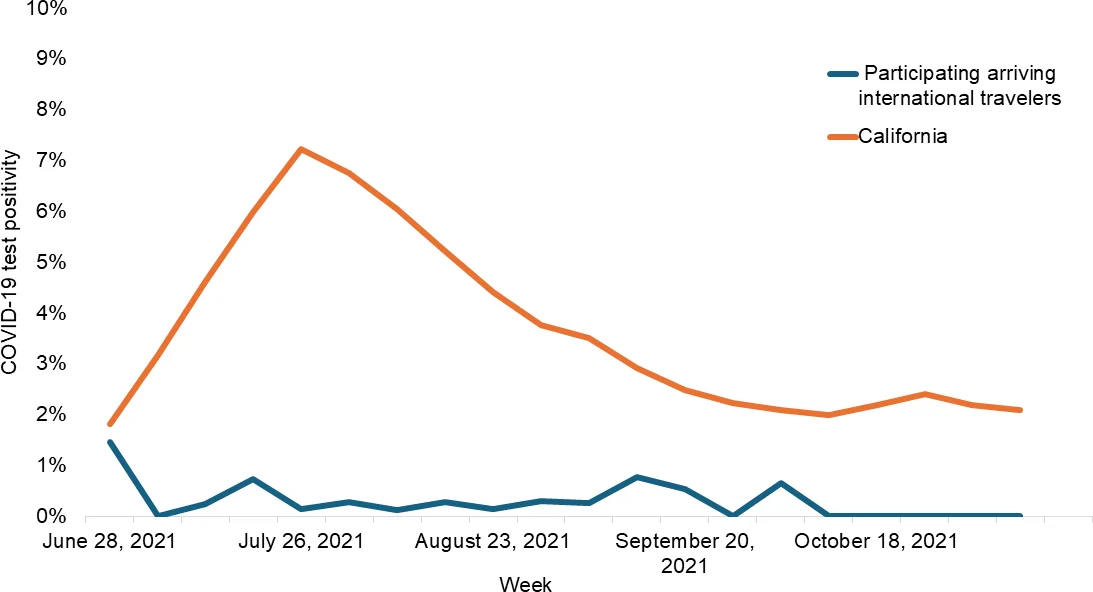
Participating arriving international travelers and California COVID-19 test positivity by week from June 29, 2021, to November 16, 2021 [18]. Blue line: COVID-19 test positivity for participating international travelers arriving at San Francisco International Airport. Orange line:COVID-19 test positivity for all of California

**Figure 5.**
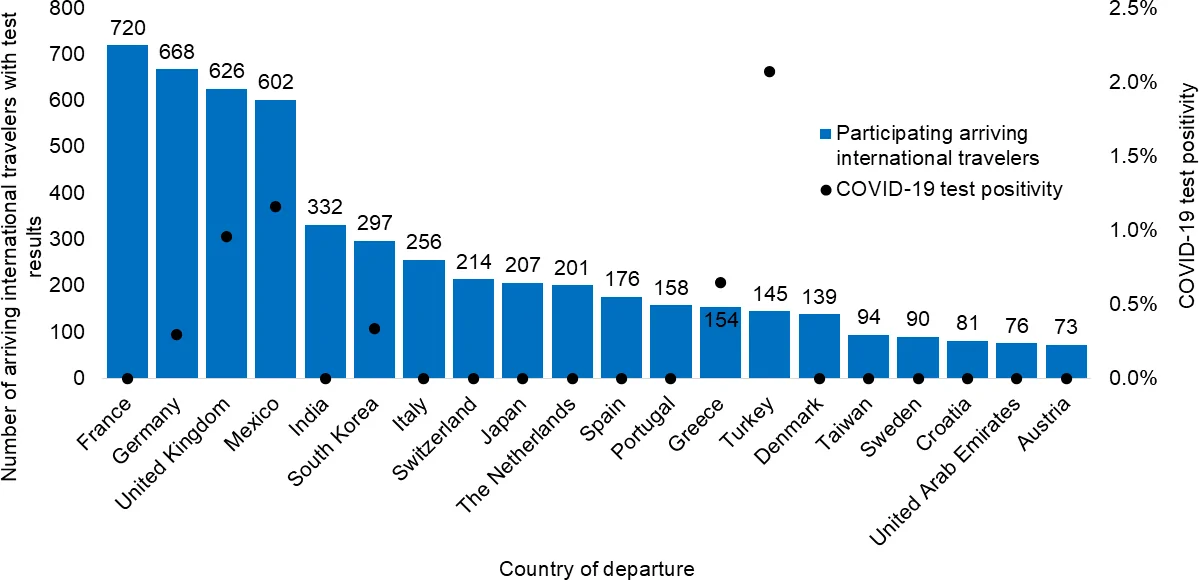
COVID-19 test positivity for arriving international traveler participants by country of departure. The blue bars represent the number of participating arriving international travelers to San Francisco International Airport by country of departure from June 29, 2021, to November 16, 2021. The black dots represents the participant COVID-19 test positivity by country of departure. The top 20 countries by number of participating arriving international travelers with test results were included.

During phase 1, a total of 28.6% (4556/15,932) of arriving international travelers reported 1 COVID-19 antigen test result, and 19.3% (3067/15,932) reported 2 antigen test results (Table 2). In total, there were 7499 antigen tests reported, 99.7% (n=7474) had a result, and 0.4% (31/7474) of tests were positive. Among those who tested positive, 67.7% (21/31) tested positive on the first antigen test administered. In phase 1, a total of 106 COVID-19 molecular tests were sent to the laboratory; 89.6% (n=95) had a result, and 12.6% (12/95) were positive. Among arriving international travelers who submitted molecular COVID-19 tests, 75% (72/96) had COVID-19 symptoms. All those who tested positive with a molecular COVID-19 test had symptoms (Table 2).

During phase 2, a total of 17.8% (549/3076) of participants sent molecular test specimens to the laboratory, 93.1% (511/549) of tests produced a result, and 0.4% (2/511) of tests were positive (Table 2). The 2 arriving international travelers who tested positive had symptoms of COVID-19. Among arriving international travelers who were tested, 8.8% (45/511) were symptomatic at the time of the test; 4.4% (2/45) of symptomatic participants tested positive. Of those who tested negative, 8.4% (43/509) had COVID-19 symptoms (Table 2).

### SARS-CoV-2 Sequencing Results

Of the 14 positive molecular tests during the pilot, 10 (71.4%) were successfully sequenced and assigned a SARS-CoV-2 lineage. All 10 samples were found to belong to the SARS-CoV-2 Delta B.1.617.2 variant, which was the most prevalent SARS-CoV-2 variant in California and worldwide at the time.

## Discussion

### Principal Findings

A two-phased pilot program consisting of diagnostic testing and SARS-CoV-2 genomic sequencing of arriving international travelers was implemented at SFO from June 2021 to November 2021. This pilot program represented the first arriving international traveler COVID-19 testing and SARS-CoV-2 WGS program in the United States and demonstrated that voluntary testing of arriving international travelers for COVID-19 and subsequent SARS-CoV-2 WGS is operationally and logistically feasible. Lessons learned during this pilot informed subsequent iterations of California’s testing and genomic surveillance system and strengthened public health partnerships across multiple agencies and private sector collaborators [13,19].

This pilot program showed that an airport-based voluntary traveler diagnostic testing and SARS-CoV-2 genomic surveillance program is feasible. The program voluntarily enrolled 2.1% (19,008/892,558) of all arriving international travelers at SFO. If all travelers who enrolled at the airport completed specimen collection, and assuming a test positivity of 0.7%, the pilot would have collected 133 SARS-CoV-2 sequences, or approximately 30 SARS-CoV-2 sequences per month. At this level of participation, the monthly variant detection threshold in arriving international travelers would be approximately 10% at 95% confidence [20]. This detection threshold from arriving international traveler voluntary testing would likely be useful to detect and characterize emerging new SARS-CoV-2 variants in travelers arriving to California.

The program rapidly built partnerships with federal, state, and local public health agencies; airport and airline authorities; immigration officials; laboratory and informatics vendors; scientific partners; and the media through multiple meetings and outreach efforts. These collaborations enabled timely launch, ongoing refinement based on stakeholder feedback, and integration of SARS-CoV-2 genomic data into California COVIDNet for statewide variant monitoring and provided statewide situational awareness [13]. California COVIDNet facilitated meetings with scientific, health care, and governmental experts across the state on a regular basis to give feedback on SARS-CoV-2 genomic surveillance efforts, including this program [13]. The pilot also developed test reporting mechanisms to enable local public health response if necessary. These mechanisms were developed based on state and local public health official feedback.

Arriving international travelers from 133 countries enrolled in this pilot program, 0.7% (33/5038) tested positive for COVID-19 despite predeparture testing, and 10 SARS-CoV-2 sequences were identified. The pilot program identified the SARS-CoV-2 Delta B.1.617.2 lineage in arriving international travelers, which was the most prevalent SARS-CoV-2 variant in California and worldwide at the time. While this pilot program established that an airport-based traveler testing workflow can be implemented successfully, difficulties with at-home specimen collection and reporting resulted in few positive specimens available for sequencing. As a result, the pilot did not demonstrate effectiveness in detecting emerging or newly introduced SARS-CoV-2 variants. Low testing completion rates of enrolled participants (less than 30% across 2 phases) were largely related to relying on arriving international travelers to complete testing and reporting at home. Low completion of at-home testing and reporting produced few positive specimens for WGS, and two-thirds of arriving international travelers who tested positive with an antigen test (20/30, 66.7%) did not have sequenced specimens. This was likely related to the difficulty of shipping a home-collected specimen to the laboratory, as well as the fact that 7.1% (47/655) of molecular specimens arrived at the laboratory too late to be tested or were inadequate specimens, leading to lost opportunities for sequencing [10,14].

The overwhelming majority of arriving international travelers tested negative for COVID-19, but there were still detections despite predeparture testing, and travelers were likely infectious during travel, highlighting infectious risks for all travelers. Participating arriving international traveler COVID-19 test positivity was lower than the test positivity for the state of California. In a study of arriving international travelers in Qatar, where a similar predeparture testing policy was in place, travelers had a similar COVID-19 test positivity to that in this study [21]. The CDC arriving international traveler predeparture COVID-19 testing requirement likely contributed to the low traveler COVID-19 test positivity [5]. Participating arriving international traveler COVID-19 test positivity varied by country of departure (Figure 5).

From phase 2 of this study, we learned that many travelers (45/511, 8.8% of participating arriving international travelers; Table 2) had symptoms of a respiratory illness during travel. Most people in phase 2 with symptoms consistent with a respiratory illness tested negative for COVID-19 (43/45, 95.6%). This highlights the utility of testing for more than just COVID-19 to determine the epidemiology of respiratory illnesses in arriving international travelers and how infections can spread via international travel.

Participating arriving international traveler vaccination rates were higher than the overall California vaccination rate and generally higher than the overall rate for departure countries during the period of the study [18]. Participant vaccination rates increased over time (Figure 3 [18]). There was variation in arriving international traveler vaccination rates by country of departure that appeared to reflect the overall vaccination rate in each country (Multimedia Appendices 2 and 3). The CDC order requiring noncitizens traveling to the United States for nonimmigration purposes to complete an initial vaccination series against COVID-19 on October 25, 2021, may have increased vaccination rates among participating arriving international travelers during program operations [7]. Some unvaccinated participating arriving international travelers were interested in getting vaccinated upon arrival at SFO when presented with the opportunity. Determining international traveler vaccination status may enable public health programs to promote vaccination for arriving international travelers and target vaccination to passengers arriving from countries where vaccination rates are lower and disease burden is higher. In future pandemics, vaccine efficacy may vary based on the viral variant.

### Lessons for Future Traveler-Based Pathogen Genomic Surveillance

Multiple operational insights emerged. Completing enrollment and specimen collection at the airport likely would markedly increase testing completion and sequencing yield. Self-collection and self-reporting at home decreased compliance and raised program costs. Integrating diagnostic testing and genomic surveillance into one specimen collection would ease participation by eliminating multiple specimen collections and reducing costs. Providing testing for free likely encouraged arriving international traveler participation. Minimizing the time for arriving international travelers to participate was important to encourage voluntary participation. Testing passengers using a point-of-care test at the airport with results available in 15 to 30 minutes could lead to behavior change that could reduce ongoing transmission from arriving international travelers to residents instead of using a test with a longer turnaround time or a test completed at home. This could be done with a low-cost COVID-19 antigen test, which likely has sufficient sensitivity to COVID-19 (approximately 85% for the BinaxNOW COVID-19 antigen test) [22]. While molecular testing may have higher sensitivity at detecting low levels of the virus, WGS is less likely to be successful with lower concentrations of SARS-CoV-2, and a higher-sensitivity molecular test may not lead to a significant increase in successful sequencing [19]. Using separate laboratories for diagnostic testing and sequencing lengthened turnaround times and complicated logistics as samples needed to be shipped multiple times. WGS from positive point-of-care test specimens may ease logistics as samples would only need to be shipped once from the collection site to the sequencing laboratory, resulting in a shorter turnaround time. These lessons have already informed improvements in California’s SARS-CoV-2 genomic surveillance program [19].

### Comparison to Other Traveler Pathogen Genomic Surveillance Programs

This pilot was conducted shortly before the CDC began its traveler genomic surveillance program in 2021 at US airports [23]. The CDC program did not perform diagnostic testing of travelers and focused on global SARS-CoV-2 genomic surveillance using a pooled sequencing strategy. Pooled sequencing does not provide data on arriving international traveler test positivity, does not provide individual participant diagnostic test results, and does not allow for local public health response. In contrast, the pilot program herein described is an example of how diagnostic testing and arriving international traveler vaccination status could be integrated into traveler respiratory virus genomic surveillance, which may have the benefit of increasing voluntary arriving international traveler participation, as well as potentially decreasing ongoing transmission from arriving international travelers and enabling local public health response for individuals who test positive.

### Limitations

There were numerous limitations of this pilot program. Arriving international traveler testing completion rates were low (5038/19,008, 26.5% of enrolled participants), leading to missed opportunities for obtaining SARS-CoV-2 sequences, decreasing program effectiveness in identifying emerging SARS-CoV-2 variants in arriving international travelers. Arriving international traveler participation was also limited due to pilot operating hours not including all flights. Language barriers or difficulty using online user interfaces may have also impacted participation completion. In addition, unsupervised anterior nasal self-swabbing may have resulted in insufficient samples, leading to false-negative tests and missed opportunities for WGS. Self-reported test results in phase 1 may be biased, possibly due to under- or overreporting of positive results relative to the true rate. Participating arriving international travelers’ COVID-19 test positivity may not be representative of the COVID-19 test positivity of travelers arriving at SFO during the study period. These factors collectively limited the program’s ability to detect emerging SARS-CoV-2 variants.

### Conclusions

This pilot program demonstrated the feasibility of airport-based traveler respiratory virus diagnostic testing and genomic surveillance. The program highlights the strength of state public health department response across multiple disciplines, including infectious diseases, epidemiology, and health communication, to improve situational awareness and public health response in the face of a global pandemic. Because of the high likelihood of future global pandemics, systems are needed to improve global situational awareness, and major international airports allow localities to efficiently access global populations where disease epidemiology, transmissibility, clinical severity, and vaccine and therapeutic effectiveness may be different from local conditions, particularly when large gaps in global surveillance exist. Despite limitations in participant test completion and sequencing yield, the program generated important operational lessons and contributed to California’s genomic surveillance infrastructure [19]. Continued advancement of traveler-based surveillance is warranted to strengthen early detection, situational awareness, and pandemic preparedness.

## Supplementary material

10.2196/96414 Multimedia Appendix 1 Participating arriving international traveler COVID-19 vaccination status by country of departure: percentage of participating arriving international travelers at San Francisco International Airport who completed a full COVID-19 vaccine series by country of departure for the top 20 participant countries from June 29, 2021, to November 16, 2021.

10.2196/96414 Multimedia Appendix 2 Participating arriving international traveler COVID-19 vaccination status by country of departure and week: percentage of participating arriving international travelers at San Francisco International Airport who completed a full COVID-19 vaccination series by country of departure from select countries by week from June 29, 2021, to November 16, 2021.

10.2196/96414 Multimedia Appendix 3 Participating arriving international traveler COVID-19 vaccination status compared to departure country vaccination status: percentage of arriving international traveler participants at San Francisco International Airport who completed a full COVID-19 vaccination series by country of departure (blue line) compared to the reported percentage of completed COVID-19 vaccination series for the same country (orange line) weekly from June 29, 2021, to November 16, 2021 [3].

## Abbreviations

CDC: Centers for Disease Control and Prevention
CDPH: California Department of Public Health
HIPAA: Health Insurance Portability and Accountability Act
WGS: whole-genome sequencing
